# Influence of Demographics and Scan Time on MK6240 Off‐Target Signal and Reference Region Selection

**DOI:** 10.1002/alz70856_100424

**Published:** 2025-12-25

**Authors:** Anthonin Reilhac, Tammie L.S. Benzinger, Kristin R Wildsmith, Brian A. Gordon, Shaney Flores, Michael C. Irizarry, Larisa Reyderman, Arnaud Charil

**Affiliations:** ^1^ Eisai Inc., Nutley, NJ, USA; ^2^ Washington University School of Medicine in St. Louis, St. Louis, MO, USA; ^3^ Washington University School of Medicine, St. Louis, MO, USA

## Abstract

**Background:**

The Tau PET tracer MK6240 occasionally exhibits off‐target signal (OTS) in the meninges, bone, and other non‐brain structures, affecting both target and reference regions when computing SUVr values. This study investigates the influence of demographics and scan time on MK6240 OTS and assesses how the choice of reference region impacts diagnostic separation. Our goal is to improve the accuracy of MK6240 PET imaging by identifying factors contributing to OTS and potential mitigation strategies.

**Method:**

We analyzed 764 baseline MK6240 PET scans from 524 healthy controls (HC), 120 mild cognitive impairment (MCI), and 120 Alzheimer's disease (AD) participants, along with 184 follow‐up scans from 149 HC, 20 MCI, and 15 AD participants from the Lantheus database. Each scan was motion‐corrected, resampled in standardized MNI space, and harmonized for spatial resolution using an in‐house developed pipeline. SUVr images were generated using three different reference regions: inferior cerebellar gray matter (GM), eroded centrum semi‐ovale (to avoid GM contamination), and the pons. OTS severity was visually rated on a 5‐point scale (0: no OTS to 4: high OTS). Statistical comparisons were made between OTS ratings and scan time, age, gender, amyloid status, and cortical tau SUVr. Performance of the reference regions was evaluated using cross‐sectional group comparisons (HC Ab‐ vs. HC Ab+, HC Ab+ vs MCI, and MCI vs AD), longitudinal changes for all groups, and correlations with cognition assessed with the MMSE.

**Result:**

Preliminary results indicate that OTS ratings are negatively correlated with age, with younger participants showing more OTS, and positively correlated with scan time, with higher OTS observed later in the afternoon. Females exhibited higher OTS than males. No correlation was found between OTS ratings and amyloid status or cortical tau SUVr. Cross‐sectional group comparisons, MMSE correlations, and longitudinal analyses suggest that the pons is a more reliable reference region than the cerebellum.

**Conclusion:**

These findings highlight the influence of demographic factors and scan time on MK6240 PET OTS and suggest that the pons may be a more reliable reference region than the cerebellum, enhancing diagnostic separation.

